# 

**Published:** 1887-11

**Authors:** 


WHOLE NUMBER CCCXXVIII.

### NOVEMBER, 1887.

NUMBER 4. VOLUME XXVIII.

### The Buffalo Medical and Surgical Journal

ESTABLISHED YEAR 1844.

EDITORS:

THOS. LOTHROP, M. D. A. R. DAVIDSON, M. D.

ASSOCIATE EDITORS:

FLOYD S. CREGO, M. D. CARLTON C. FREDERICK, M

FRED. R. CAMPBELL, M. D. FRANK H. POTTER, M. D.

EDWARD B. ANGELL, M. D., Rochester, N. Y.

